# The efficacy and safety of endostar combined with taxane-based regimens for HER-2-negative metastatic breast cancer patients

**DOI:** 10.18632/oncotarget.8967

**Published:** 2016-04-25

**Authors:** Weiwei Huang, Jian Liu, Fan Wu, Kan Chen, Nani Li, Yi Hong, Cheng Huang, Hongyu Zhen, Lin Lin

**Affiliations:** ^1^ Department of Medical Oncology, Fujian Provincial Cancer Hospital, the Teaching Hospital of Fujian Medical University, the Teaching Hospital of Fujian University of Traditional Chinese Medicine, Fuzhou 350014, China

**Keywords:** endostar, taxanes, HER-2 negative, metastatic breast cancer

## Abstract

The purpose of the present study was to prospectively evaluate the efficacy and safety of endostar, a recombinant product of endostatin, combined with taxane-based regimens for HER-2 negative metastatic breast cancer (MBC) patients. Women with ages between 18–70 years with histologically confirmed MBC documented as HER-2-negative were included. Endostar was administered at 7.5 mg/m^2^, d1–14, q21d and was continued until progressive disease, unacceptable toxicity, consent withdrawal, or completion of 24 months of endostar, whichever came first. Taxane-based chemotherapy was continued until progressive disease, unacceptable toxicity, consent withdrawal, or up to 8 cycles. The primary endpoint was overall response rate (ORR). Fifty-seven patients were recruited. The ORRs for the whole population, first-, second-, and third-line therapy or beyond were 68.4%, 79.3%, 54.5%, and 16.7%, respectively. The median PFS was 10.8 (8.0–12.1) months, yet the median OS was still not attained. For the patients receiving first-, second-, and third-line therapy or beyond, median PFS was 11.9, 7.5, and 7.4 months, respectively (P=0.048). No significant difference in median PFS between hormonal receptor-positive and -negative patients was observed. The most common drug-related grade 3–4 hematologic toxicities were neutropenia (80.7%) and leukopenia (77.2%). Six (10.5%) patients experienced febrile neutropenia. The most frequent drug-related grade 3–4 non-hematologic toxicities were liver dysfunction (10.5%) and peripheral neurotoxicity (8.8%). No treatment-related deaths were reported. We conclude that Endostar combined with taxane-based regimens may be effective and safe for the treatment of HER-2-negative MBC. However, further investigations on its long-term efficacy and toxicity are warranted.

## INTRODUCTION

Despite significant improvements in the treatment of early-stage breast cancer, approximately 20%–40% of patients develop relapse and distant recurrence, whereas some present with de novo metastatic disease. The treatment intent in metastatic breast cancer (MBC) patients is primarily noncurative, i.e., palliative, in nature. Overall, 5-year survival rates are reported to approximate 24% [1]. Therefore, treatments that provide more clinical benefit among these patients, especially the human epidermal growth factor receptor 2 (HER-2)-negative subgroup, which lacks anti-HER-2 therapies, will continue to be sought.

Antiangiogenesis is a promising antitumor strategy that inhibits tumor vascular formation to suppress tumor growth. Several antiangiogenic targeting molecules have been tested and are now used for cancer treatment. However, bevacizumab, as a monoclonal antibody directed against circulating vascular endothelial growth factor (VEGF), was removed from MBC indication in December 2010 based on the poor results of cost-benefit analysis. In addition, neither sunitinib nor sorafenib, which are anti-VEGF receptor (VEGFR) tyrosine kinase inhibitors (TKIs), had a major impact in MBC [2]. It is thus imperative to develop another strategy that inhibits angiogenesis in breast cancer.

Endostar is a recombinant product of endostatin, which is an endogenous inhibitor of angiogenesis [3]. Animal studies have shown that endostatin is capable of blocking the proliferation and organization of endothelial cells into new blood vessels *in vitro* and inhibiting angiogenesis and growth of both primary tumors and secondary metastasis [3]. Although previous clinical studies showed that endostatin alone did not result in significant improvements in cancer progression, it can be beneficial when combined with other chemotherapies. A phase III clinical trial on 493 stage IIIB and IV non-small cell lung carcinoma (NSCLC) patients showed that the addition of endostar to a vinorelbine/cisplatin regimen resulted in significant and clinically meaningful improvement in response rate, median time to progression, and clinical benefit rate compared to the chemotherapeutic regimen alone [4]. Regarding breast cancer, *in vivo* studies showed that the combination of paclitaxel and P125A-endostatin inhibited mammary cancer growth, delayed the onset of multifocal mammary adenocarcinomas, decreased tumor angiogenesis, increased the survival of treated mice in the prevention model, and inhibited lung and lymph node metastasis in the intervention model [5]. Sun et al. also reported that the tumor-inhibiting effect of the paclitaxel-cisplatin (TP) regimen combined with recombinant human endostatin on breast cancer is better than that of the TP regimen alone in xenograft-bearing nude mice [6]. Moreover, in a prospective, randomized, controlled, phase II neoadjuvant trial, the combination of rh-endostatin with chemotherapy produced a higher tumor response rate without increasing toxicity in breast cancer patients [7]. Considering these promising data, this prospective study was conducted to evaluate the efficacy and safety of endostar combined with taxane-based regimens for HER-2-negative MBC patients.

## RESULTS

### Patients

Between January 2011 and December 2014, 57 patients with invasive ductal carcinoma were recruited. The characteristics of the patients are listed in Table 1. The median age was 50 years (range: 34–69 years). Fifty (87.7%) patients were hormonal receptor-positive. Twenty-nine (50.9%) patients had ≥ 3 metastatic organ sites, and visceral involvement was noted in 48 (84.2%) patients. Twenty-nine (50.9%) patients had no previous chemotherapies for metastatic disease.

**Table 1:** Patient characteristics (N=57)

Characteristics	Number	%
Age, years		
Median	50	
Range	34-69	
ECOG status		
0	28	49.1
1	29	50.9
Hormonal receptor status		
Positive	50	87.7
Negative	7	12.3
Menstruation status		
Pre- or Peri- menstruation	23	40.4
Post menstruation	34	59.6
Metastatic sites		
Liver	21	36.8
Lung	30	52.6
Lymph nodes	9	15.8
Bone	20	35.1
Visceral	48	84.2
Non-visceral only	9	15.8
No. of metastatic sites		
1	10	17.5
2	18	31.6
≥ 3	29	50.9
Previous surgical treatment of breast cancer		
Yes	49	86.0
No	8	14.0
Number of previous lines of chemotherapies		
None	29	50.9
1	22	38.6
≥ 2	6	10.5
Previous chemo drugs		
Adjuvant setting		
Anthracycline	39	68.4
Taxane	34	59.6
Both	25	43.9
Metastatic setting		
Anthracycline	7	12.3
Taxane	15	26.3
Both	5	8.8

### Efficacy

The median number of treatment cycles was 4 (range: 2–8 cycles). Treatment efficacy is summarized in Table 2. The overall response rates (ORRs) for the whole population, first-, second-, and third-line therapy or beyond were 68.4%, 79.3%, 54.5%, and 16.7%, respectively. Although not statistically significant (P=0.542), a numerically higher response rate (78.6%) was observed in patients who had not received taxanes, either in the neoadjuvant/adjuvant setting or metastatic setting, compared to the 65.1% observed in those pretreated with taxanes. Objective response was not associated with hormonal receptor status, visceral involvement, liver metastasis, lung metastasis, and the number of metastatic sites (data not shown).

**Table 2:** Summary of treatment efficacy

Variable	N	ORR (%)	P value	Median PFS (Months, 95%CI)	P value	Median OS (Months, 95%CI)	P value
Whole group	57	68.4		10.8 (8.0–12.1)		not reached	
Hormonal receptor status			0.802		0.461		0.653
Positive	50	68.0		11.2 (9.3–12.8)		not reached	
Negative	7	71.4		9.8 (9.1–10.5)		22.5	
Lines of treatment			0.009		0.048		0.218
First-line	29	79.3		11.9 (10.0–13.1)		not reached	
Second-line	22	54.5		7.5 (4.3–11.4)		23.1 (13.2–33.0)	
Third- or more line	6	16.7		7.4 (4.0–11.0)		24.2	
Previous taxanes			0.542		0.058		—
Yes	43	65.1		8.9 (6.0–11.3)		16.8 (11.2–22.5)	
No	14	78.6		11.4 (9.0–13.1)		not reached	

Abbreviations: ORR: overall response rate; PFS: progression free survival; OS: overall survival; CI: confidence interval.

After a median follow-up of 18.2 months, the median progression-free survival (PFS) was 10.8 (8.0–12.1) months, yet the median overall survival (OS) was still not achieved. The patients not pretreated with taxanes showed a trend of longer median PFS compared to those pretreated with taxanes (P=0.058). For the patients receiving first-, second-, and third-line therapy or beyond, median PFS was 11.9, 7.5, and 7.4 months, respectively (P=0.048). No statistically significant difference in median PFS between hormonal receptor-positive and -negative patients was observed.

### Toxicity

The toxicity profile of the combination treatment was acceptable and manageable. The most common drug-related adverse events (AEs) are presented in Table 3. The most common grade 3–4 hematologic toxicities were neutropenia (80.7%) and leukopenia (77.2%). Six (10.5%) patients experienced febrile neutropenia. The most frequent grade 3–4 non-hematologic toxicities were liver dysfunction (10.5%) and peripheral neurotoxicity (8.8%). The endostar-related or -possibly-related AEs included grade 1–2 arrhythmia (2 patients) and grade 1–2 hypertension (3 patients), which were well controlled with antiarrhythmic drugs and myocardial nutritional medicine, and antihypertensives, respectively. No epistaxis and proteinuria were observed. Dose adjustment due to AEs was performed on 18 patients (31.6%). No treatment-related deaths were reported.

**Table 3:** Drug related adverse events (N = 57)

Adverse Event	Grade 1		Grade 2		Grade 3		Grade 4	
	Number	%	Number	%	Number	%	Number	%
Hematologic								
Leukopenia	2	3.5	11	19.3	28	49.1	16	28.1
Neutropenia	2	3.5	9	15.8	29	50.9	17	29.8
Febrile neutropenia	NA		NA		6	10.5	0	0
Anemia	11	19.3	24	42.1	5	8.8	1	1.8
Thrombocytopenia	6	10.5	9	15.8	1	1.8	0	0
Non-hematologic								
Rash	12	21.5	4	7.0	1	1.8	0	0
Peripheral neurotoxicity	16	28.1	11	19.3	5	8.8	0	0
Alopecia	31	54.4	26	45.6	NA		NA	
Fatigue	24	42.1	3	5.3	0	0	NA	
Nausea	27	47.3	11	19.3	1	1.8	NA	
Vomiting	21	36.8	10	17.5	1	1.8	0	0
Diarrhea	2	3.5	4	7.0	2	3.5	0	0
Constipation	8	14.0	7	12.3	0	0	0	0
Liver dysfunction	10	17.5	4	7.0	6	10.5	0	0
Arrhythmia	1	1.8	1	1.8	0	0	0	0
Hypertension	2	3.5	1	1.8	0	0	0	0

## DISCUSSION

To our knowledge, this is the first study to prospectively investigate the efficacy and toxicity of endostar combined with taxane-based regimens for HER-2-negative MBC patients. Actually, as a recombinant product of endostatin, the antiangiogenesis mechanism of endostar involves its broad spectrum of activity that is focused on preventing angiogenesis, including interfering with TNF-alpha activation of JNK, inhibiting cyclin D1, blocking FGF signal transduction, and suppressing Ras/Raf kinases and decreasing ERK-1 and p38 activity [10–13]. Our results demonstrated an ORR of 68.4% in patients with HER-2-negative MBC, which met its primary endpoint, with a 60% ORR for the whole population.

Antiangiogenic approaches represent a promising new strategy for the treatment of cancer. Although the VEGF signaling pathway, a well-recognized angiogenic factor playing a crucial role in regulating tumor angiogenesis [14] and normal vascular development [15, 16], is considered as a good antitumor target, the routine role of bevacizumab in the management of HER-2-negative MBC remains controversial. Meta-analysis has shown that its addition to first-line chemotherapy in MBC patients is associated with an increase in ORR and prolonged PFS in three randomized phase III trials, namely, E2100, AVADO, and RIBBON-1 [17]. Even in the second-line setting (RIBBON-2), significant improvements in PFS with bevacizumab were observed (median 7.2 *vs*. 5.1 months for chemotherapy plus bevacizumab *vs*. chemotherapy alone; P=0.0072) [18]. Although the FDA has removed the MBC indication from bevacizumab, taxane plus bevacizumab is still recommended by various guidelines, including the National Comprehensive Cancer Network (NCCN) guidelines, and is continued to be used in clinical practice especially for those with immediately life-threatening disease or severe symptoms, thereby suggesting that antiangiogenic approaches are still considered in breast cancer treatment and thus deserve further assessment.

In the present study, the ORR and PFS of the 29 patients receiving the combination therapy as first-line treatment was 79.3% and 11.9 months, respectively, which were markedly higher than those of chemotherapy plus bevacizumab in E2100, AVADO, and RIBBON-1 [17] and consistent with the results of a neoadjuvant trial showing that the ORR significantly increased from 67.7% to 90.9% when combined with rh-endostatin [7]. Even in second- and third-line or more settings, the PFS of this combination still reached more than 7 months, which is similar to that reported in RIBBON-2 [18]. Our data also showed that this combination is effective in patients pretreated with taxanes, with an ORR of 65.1% and a PFS of 8.9 months. No statistically significant associations of ORR or median PFS with hormonal receptor status were observed, indicating that the combination might be equally effective between luminal and triple-negative subtypes. Considering its promising efficacy, this combination regimen is thus worthy of further investigation in a randomized phase III study.

With regard to safety, the combination of endostar with taxane-based regimens did not result in any significant changes to the AE profile of taxane-based regimens nor did it increase fatal toxicities. Different from the observation of increased rates of hypertension (e.g., in E2100 [14.8%] and RIBBON-1 [8.9%]) and proteinuria (e.g., in E2100 [3.5%] and RIBBON-1 [3.9%]) in prior studies with bevacizumab [19], the incidence of hypertension in our study was only 5.3%, and no proteinuria was detected.

The present study has several limitations. First, the sample size was relatively small. Second, the study consisted of a heterogeneous patient population as patients received endostar combined with taxane-based regimens in different treatment lines, which resulted in some uncertainty regarding the treatment effect. The heterogeneity among different taxane-containing chemotherapies should also be noted and suggested that the results be interpreted with caution. However, these could provide some useful information to design a randomized study focusing on a determined treatment line and a determined regimen. Lastly, tumor markers were not measured. Despite of these limitations, the present study is the first to reveal the potential efficacy and toxicity of endostar in MBC.

In conclusion, this pilot clinical trial provides evidence that endostar enhances the antitumor effects of taxane-based chemotherapy in MBC patients with well-tolerated toxicities. Furthermore, the preliminary results support the setup of a larger sample, multicenter, randomized, phase III clinical trial to provide a definitive validation for the use of endostar in future strategy against HER-2-negative MBC.

## PATIENTS AND METHODS

### Patients

HER-2-negative status was assessed by IHC and/or fluorescence in situ hybridization (FISH). HER-2-negative was defined as no staining by IHC, and HER-2 gene amplification by FISH was performed for those cases with scores of 1+ and 2+ by IHC and confirmed absence of gene amplification by FISH. Hormonal receptor -negative was defined as < 1% positive tumor cells immunohistochemical nuclear staining of both estrogen receptor (ER) and progesterone receptor (PR) according to ASCO guidelines. Therapy with a taxane as part of adjuvant or neoadjuvant therapy was allowed, but should have been completed at least 12 months before study entry. Prior taxanes in the metastatic setting were also permitted when these were completed at least 3 months before study enrolment. Patients must have at least 1 prior line of endocrine therapy when hormone receptor was positive, at least one measurable disease according to RECIST 1.1 criteria, a life expectancy of no less than 3 months, Eastern Cooperative Oncology Group (ECOG) performance status ≤ 1 and adequate hematologic, renal, and hepatic function, as indicated by hemoglobin ≥ 9g /dL, absolute neutrophil count (ANC) ≥ 1.5 × 10^9^/L, platelet count ≥75 × 10^9^/L, total serum bilirubin ≤1.5 × upper limit of normal (ULN), AST/ALT ≤ 2.5 × ULN (≤ 5× ULN in case of liver metastases), and serum creatinine ≤ 1.0 × ULN (calculated creatinine clearance ≥ 50 mL/min).

Patients were excluded when they regularly received corticosteroids or immunosuppressive medications, had clinical evidence of brain metastasis or unhealed wound, or had preexisting peripheral neuropathy higher than grade 1.

The study was approved by the Fujian Provincial Cancer Hospital Ethic Committee for Clinical Investigation. The study was conducted in accordance with the Declaration of Helsinki. Written informed consent was obtained from all patients prior to enrollment.

### Treatment

Taxane-based chemotherapy choices included paclitaxel 80 mg/m^2^ qw (12.3%); ABX 130 mg/m^2^ qw (43.9%); GT: paclitaxel 175 mg/m^2^ d1+gemcitabine 1,000 mg/m^2^ d1d8 q21d (14.0%); or XT: Docetaxel 75 mg/m^2^ d1+capecitabine 1,000 mg/m^2^ bid d1–14 q21d (29.8%). Chemotherapy was continued until progressive disease, unacceptable toxicity, consent withdrawal, or up to 8 cycles, whichever came first. Dose modifications were made if grade 4 neutropenia lasted longer than 3 days, grade 3-4 thrombocytopenia, febrile neutropenia and/or grade 3 non-hematological toxicity (except alopecia and inadequately treated nausea/vomiting). In this case, treatment was interrupted for up to 2 weeks until resolution to grade ≤ 2, and doses of administered chemo drugs were reduced permanently by 20%–25% in subsequent cycles. When the observed toxicity did not resolve after 2 weeks or dose modifications occurred more than twice, the patient was withdrawn from the study. In addition, patients who experienced any grade 4 non-hematological toxicity were withdrawn from the study. Administration of prophylactic G-CSF was not permitted in the study.

Endostar was administered at 7.5 mg/m^2^, d1–14, q21d and was continued until progressive disease, unacceptable toxicity, consent withdrawal, or completion of 24 months of endostar, whichever came first. When chemotherapy was discontinued before progressive disease, the patients continued treatment with endostar monotherapy. No reduction in endostar dose was permitted.

### Assessment

Pretreatment assessment included a detailed medical history, physical examination, routine laboratory tests, and assessment of performance status. Laboratory evaluation included a routine blood count, biochemistry including electrolytes, renal and liver function tests, and urinalysis. AEs and concomitant medications were recorded at the end of each cycle throughout the study period until 30 days after the last dose of a study treatment was administered. Those AEs assessed by the investigators as “;possibly related” or “related” to study drug treatment were defined as the “drug-related AEs”. Toxicity was evaluated and graded according to the National Cancer Institute Common Terminology Criteria for AEs, version 4.0.

Radiographic scans (Computed Tomography scans or Magnetic Resonance Imaging) for efficacy evaluation were conducted at baseline and every two treatment cycles thereafter per RECIST 1.1 guidelines. The best overall response was reported. For patients without progress at the end of treatment, radiographic assessment was performed every 2 months within the first 6 months and every 3 months thereafter until disease progress. Survival status was assessed every 3 months after disease progress.

### Statistical methods

The primary endpoint was ORR (CR plus PR). Secondary endpoints included PFS, OS, and safety.

Sample size was calculated on the basis of Simon's two-stage minimax design [8]. The ORR of combination was hypothesized as 65% with the addition of endostar in the whole HER-2-negative MBC population compared with 45% for taxane-based regimen [9]. The design had 90% power with a type I error of α=0.05. When 14 of the first 31 patients enrolled achieved complete response [CR] or partial response [PR], the study was moved to the second stage, and when there were 30 out of a total of 54 patients responded to the treatment, the study was deemed positive and has proven the hypothesis. The sample size was calculated as 57, which included a 5% dropout rate.

All statistical analyses were conducted using SPSS version 18 (SPSS Inc., Chicago, IL). PFS was defined as the interval between inclusion and documented disease progression, or death as a result of any cause in patients with no evidence of disease progression. OS was defined as the interval between inclusion into the study and death. Safety issues, including incidence and severity of AEs, were also investigated. PFS and OS were estimated, and 95% confidence intervals were calculated by means of the Kaplan-Meier method. All P values reported were two-sided and were considered significant when <0.05.

## Abbreviations

MBC: metastatic breast cancer
HER2: human epidermal growth factor receptor 2
VEGF: vascular endothelial growth factor
TKI: tyrosine kinase inhibitors
NSCLC: non-small cell lung carcinoma
NCCN: National Comprehensive Cancer Network
FISH: fluorescence in situ hybridization
ECOG: Eastern Cooperative Oncology Group
ANC: absolute neutrophil count
ULN: upper limit of normal
AE: Adverse event
ORR: overall response rate
PFS: progression-free survival
OS: overall survival.
